# The effect of a peer-led problem-support mentor intervention on self-harm and violence in prison: An interrupted time series analysis using routinely collected prison data

**DOI:** 10.1016/j.eclinm.2020.100702

**Published:** 2021-01-15

**Authors:** Amanda E. Perry, Mitch G. Waterman, Veronica Dale, Keeley Moore, Allan House

**Affiliations:** a Department of Health Sciences, University of York, York YO10 5DD, UK; b School of Psychology, University of Leeds, Leeds LS2 9JT, UK; c HMP Wealstun, Thorpe Arch, Wetherby LS237AZ, UK; d School of Medicine, University of Leeds, Leeds LS2 9JT, UK

**Keywords:** Problem-solving therapy, Interrupted time series, Systematic review, Prison, Self-harm, Violence, Offenders

## Abstract

**Background:**

Levels of mental disorder, self-harm and violent behaviour are higher in prisons than in the community. The purpose of this study was to determine whether a brief peer-led problem-support mentor intervention could reduce the incidence of self-harm and violence in an English prison.

**Methods:**

An existing intervention was adapted using a theory of change model and eligible prisoners were trained to become problem-support mentors. Delivery of the intervention took two forms: (i) promotion of the intervention to fellow prisoners, offering support and raising awareness of the intervention but not delivering the skills and (ii) delivery of the problem-solving therapy skills to selected individual prisoners. Training and intervention adherence was measured using mentor log books. We used an Interrupted Time Series (ITS) design utilizing prison data over a 31 month period. Three ITS models and sensitivity analyses were used to address the impact across the whole prison and in the two groups by intervention delivery. Outcomes included self-harm and violent behaviour. Routine data were collected at monthly intervals 16 months pre-, 10 months during and six months post-intervention. Qualitative data measured the acceptability, feasibility, impact and sustainability of the intervention. A matched case-control study followed people after release to assess the feasibility of formal evaluation of the impact on re-offending up to 16 months.

**Findings:**

Our causal map identified that mental health and wellbeing in the prison were associated with environmental and social factors. We found a significant reduction in the incidence of self-harm for those receiving the full problem-solving therapy skills. No significant reduction was found for incidence of violent behaviour.

**Interpretation:**

Universal prison-wide strategies should consider a series of multi-level interventions to address mental health and well-being in prisons.

**Funding:**

Research Champions Fund and the Economic and Social Research Council Impact Acceleration Account Fund, University of York, UK.

**Research in Context**

**Evidence before this study**

Using Advanced Google Scholar we conducted citation searches of randomised controlled trials from 2014 to May 2020 using the search (self-harm) AND (problem-solving) to update previous systematic reviews.

**Added value of this study**

We found that mental health and well-being in prison were associated with environmental and social factors. Routinely collected data showed how a brief peer-led PSM intervention could reduce incidence of self-harm, but no significant difference was found for violent behaviour.

**Implications of all the available evidence**

Interventions for mental health and well-being in prisons should emphasise the importance of using a multi-level approach to support the reduction of self-harm and violent behaviour in prisons.

Alt-text: Unlabelled box

## 1 Introduction

The mental health of people incarcerated in prison is recognised as a worldwide public health concern [1]. People residing in prison experience higher levels of mental health problems, self-harm and anti-social violent behaviour than in the general population [2], [3], [4], [5]. Isolation and boredom link to poor mental health and can exacerbate these and other health problems [6]. In the last five years, UK prisons have reported an unprecedented rise in the incidence of violent assaults and self-harm [7,8]. The co-morbidity of these incidents is well documented [2,[9], [10], [11]]. Interpersonal violence is the 13th leading cause of disability life adjusted years in 25–49 year olds globally, and the societal costs of mental health problems therefore extend beyond prison, and those diagnosed with a mental health problem while in prison are more likely to reoffend in comparison to their counterparts [12].

Reviews [2,[13], [14], [15]] of mental health interventions in prisons identify randomised controlled trials of psychological and medical interventions, however the evidence is often based on trials of small sample sizes and poorly described intervention mechanisms. This makes it difficult to determine how interventions can be adapted or when they should be implemented [16]. Alternative research designs are therefore required to assess how interventions can help support people with mental health problems in prison [9,17]. The costs of such evaluations are large, it would therefore be useful to know whether routinely collected prison data could be used to assess intervention change. Other evaluations of routinely collected data use interrupted time series designs and mainly predominate in healthcare settings, the strengths of the design include the use of existing longitudinal data [18,19].

**Fig. 1:**
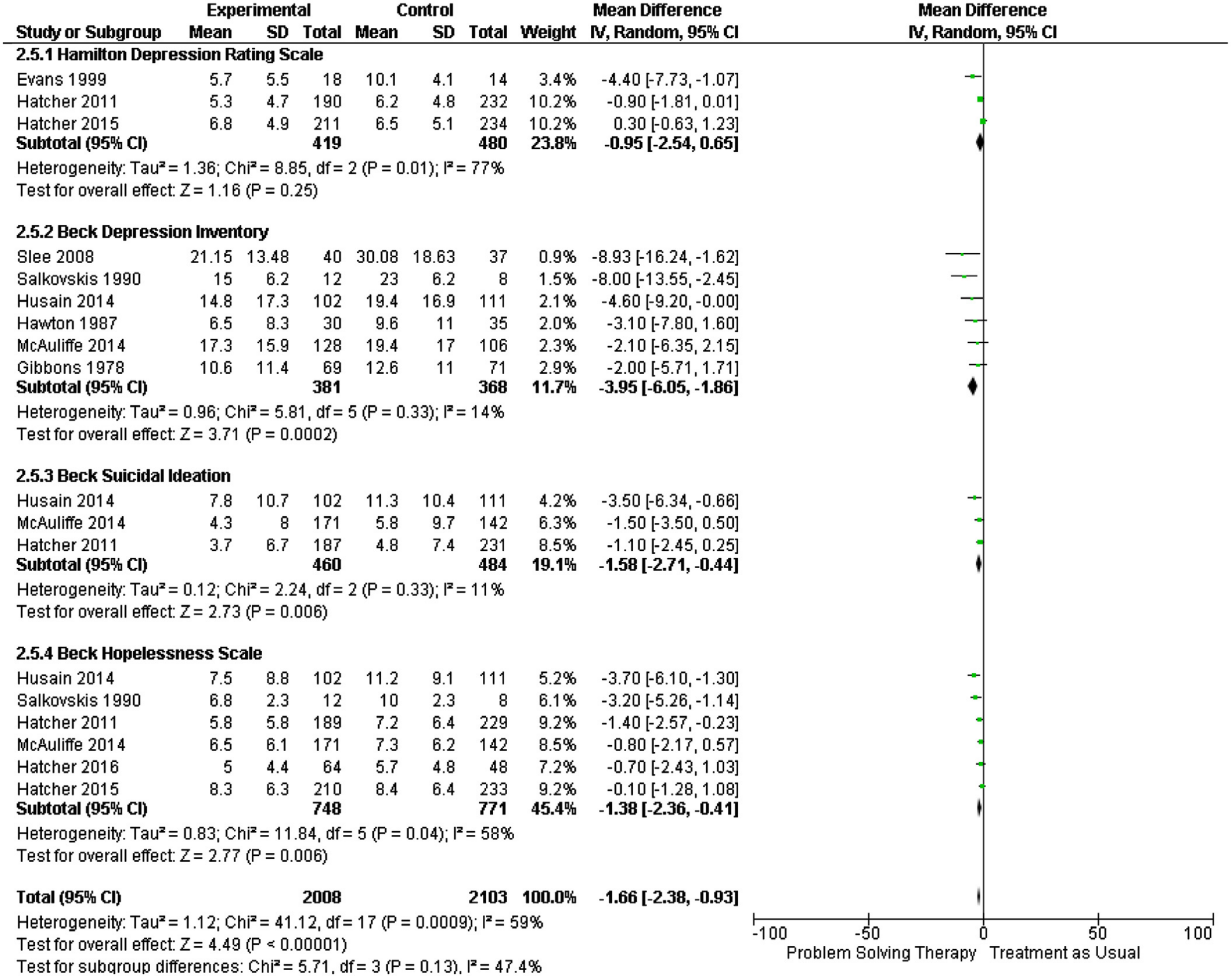
Problem solving therapy versus treatment as usual impact of depression, suicidal ideation and hopelessness at final follow-up.

**Fig. 2:**
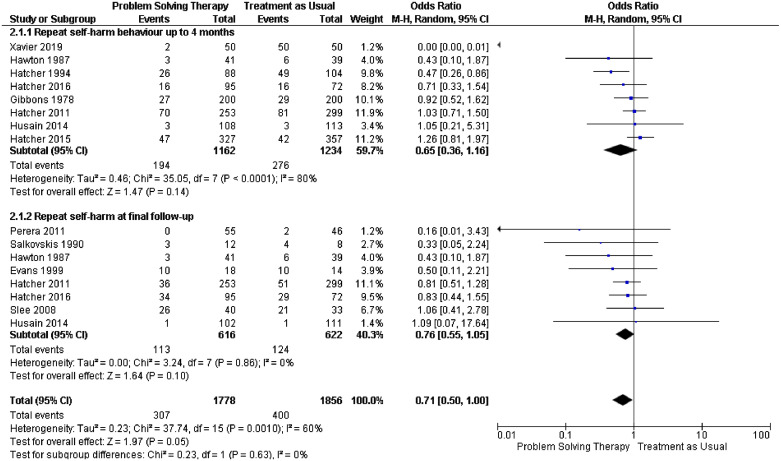
Problem solving therapy versus treatment as usual repetition of self-harm behaviour at 4 months and final follow-up.

Many people who display symptoms of depression, self-harm or violent behaviour report the main immediate cause as being problems in their lives [20], [21], [22]. Problem-solving therapy (PST) has been widely used in the community and improves outcomes of depression and allied constructs such as hopelessness [23,24]. Our new systematic review included trials where the main intervention was described as ‘problem-solving therapy’ (supplementary file Appendix A). We identified 7 new studies bringing the total of studies in the review to *n* = 24. Meta-analyses utilising data from 21/24 studies showed that PST in comparison to treatment at usual at final follow-up was found to reduce symptoms of depression (Fig. 1) using the Beck Depression Inventory, (MD −3.95, 95% CI −6.05 to −1.86), the Hamilton Depression Rating Scale (MD −0.95, 95% CI −2.54 to 0.65), hopelessness, measured by the Beck Hopelessness Scale (MD −1.38, CI 95% −2.36 to −0.41) and suicidal ideation (MD −1.58, CI 95% −1.58 to −0.44). Fig. 2 shows outcomes of repetition of self-harm at 4 months (OR 0.65, CI 95% 0.36 to 1.16) and final follow-up (OR 0.76, CI 95% 0.55 to 1.05). Such skills can be delivered by a range of professional and lay person groups and the World Health Organisation have adopted them to help those dealing with international crisis situations [25,26]. The simplicity of the skills and the ease of delivery suggests the approach may help people who experience problems in custody.

Little is known about the mechanisms underlying the impact of interventions on mental health and well-being in the prison environment [27], [28], [29], [30], [31]. Complex interventions are notoriously difficult to implement and evaluate where uncertainty about the stability of the environment exists [32], [33], [34], [35]. Audit results from 275 prisoners identified that 65% would prefer to talk to a fellow prisoner about their problems because they may have experienced similar difficulties [36]. Reviews of peer-led prison interventions have shown equivocal results in reducing risky behaviours [37]. The evidence is not clear cut and more sophisticated evaluations using robust research designs are needed to identify outcomes of effectiveness including costs [38].

We aim to fill these gaps by using an ITS to investigate the impact of a brief peer-led problem-support mentor (PSM) intervention. Our rationale encompasses the idea that support is provided by those who share similar attributes or types of experience, to promote a formalised use of PST skills [37]. The prevention model was aimed at using routinely collected prison data to evaluate the impact of an intervention to help individuals identify and deal with problems before they escalated into use of anti-social violent behaviour or self-harm [38].

## 2 Methods

### 2.1 Setting and study design

The site involved one resettlement prison housing up to 825 male adult sentenced prisoners for any form of crime except sexual offenses in the North of England. The study was divided into three stages. In stage one, we refined an existing intervention using a co-produced Theory of Change (ToC) model [27]. This was used to adapt and improve the existing training manual. In stage two, we implemented the peer-led problem-support mentor intervention. After training, the delivery of the intervention took two forms (i) promotion of the intervention to fellow prisoners offering support and raising awareness of the intervention but not delivering the skills or (ii) delivery of the problem-solving therapy (PST) skills to individual prisoners. Ethical approvals were granted by HMPPS National Research Committee through two applications [HMPPS, 2017–281] and [HMPPS 2018–355]. Written informed consent was gathered from all mentors involved in the study. We follow the Strengthening of the Reporting of Observational Studies in Epidemiology (STROBE) guide for the reporting of observational studies [39].

### 2.2 Stage one: refining the intervention using a theory of change framework

Previous studies [20,40,41] using the PST skills were co-produced with prison staff and prisoners and refined further using a ToC model [27]. Two, three-hour workshops were led by a prison program team lead (KM) and the PI (AP). The workshops were devised using the theory of change model. These were used to explore factors affecting the mental health and well-being of prisoners (environmental, social and cultural) to produce a causal map. The responses of prison staff and prisoners were separated and higher-level constructs (where six or more factors linked into one element) were used to produce the theory of change model (supplementary Appendix B). The causal map was used to identify which elements of the intervention supported mental health and well-being and the training manual was adapted to incorporate these constructs.

### 2.3 Stage two: implementation of the peer-led problem-support mentor intervention

.

### 2.4 Eligibility criteria

Participants were recruited to the role of the PSM using advertisements and staff recommendations. Participants were excluded for: (i) markers for bullying and/or violence in the previous 6 months, (ii) posing a risk to the researcher, (iii) having less than 6 months to transfer or release, (iv) being on a ‘basic’ standard of living (referring to the basic prisoner entitlements) due to problematic behaviour within the prison and were (v) excluded if they were non-English speaking.

### 2.5 Training of the PSMs

Training in PST skills was delivered by a chartered forensic psychologist (AP) and the prison administrative team. Five one-hour training sessions followed a seven step model [42]. The sessions included: (i) a general introduction to the intervention, (ii) a demonstration of the PST skills, (iii) practice of the PST skills, (iv) helping someone else to problem-solve, and (v) understanding the role of the PSM. Participants were involved in individual exercises, group work, presentations and role play. Training was followed by bi-weekly one-hour supervision sessions. After a six-week period the PSMs received a certificate and working reference. The intervention was promoted within the prison (using articles, posters, events, social media, and attendance at senior management team meetings) and nationally (https://insidetime.org/problem-solved/).

### 2.6 Data collection procedures: PSM data collection

Self-report demographic questionnaires collected data on: age, ethnicity, religion, marital status, educational background, previous and current criminal history and prison number. PSMs used paper log books to collect data on how the intervention was delivered (promotional only or delivery of the full PST skills), the type of problems they dealt with, the duration of intervention delivery, time and date of delivery, the source of the referral and any actions taken as a result of supporting the peer. Data were entered into an Excel database and collated to describe training and intervention adherence and length of engagement.

### 2.7 Routinely collected prison data

Incidents of self-harm and violence were obtained from two routinely collected databases. Violent incidents represented 18 different categories of violent and disruptive behaviour (supplementary file Appendix C). Self-harm incidents were determined by the prison Assessment Care in Custody and Teamwork (ACCT) framework which records anyone at risk of self-harm or suicidal behaviours [43].

Peers receiving each form of the intervention and the PSMs were tracked through each database using the individual prison number. The number of incidents at monthly intervals before (November 1st 2016 to February 28th 2018) during (February 26th and 13th of December 2018) and after delivery of the intervention (up until 14th June 2019).

### 2.8 Qualitative data collection

Between May and October 2018 PSMs were invited to attend small group (2–3) qualitative interviews. An independent researcher devised and conducted the interviews using a semi-structured interview topic guide. This was designed to identify (i) the experience of training and the acceptability of the training materials, (ii) the experiences of the role, and (iii) ideas for how the intervention could become sustainable.

## 3 Stage three: follow-up study

36 cases were matched to 36 controls on a ratio of 1:1. They were followed from date of first release or transfer to another prison for up to 16 months using the prison National Offender Manager Information System (p-NOMIS). PSMs were matched to a comparable control using date of release, age group, standard of living upon release, offence category and length of sentence. The outcomes were mapped into four categories: (i) released and remained in the community, (ii) released and returned or recalled to custody, (iii) prisoner remains in custody or (iv) prisoner remains in custody but transferred to another prison site.

## 4 Statistical analysis

### 4.1 PSM data analyses

Individual data were aggregated and summarised using descriptive statistics to describe the characteristics of the PSMs using an Excel database and statistical package SPSS.

### 4.2 The ITS design

An ITS analysis was used to evaluate an intervention using regression modelling intervention on incidence of pre and post intervention self-harm and violent behaviour. There were three analyses of interest. The first was the effect on the whole prison (groups one and three). The second and third analyses were defined by exposure to the intervention (promotional only or delivery of the full skills). Analyses were conducted separately for the number of self-harm and violent incidents (groups two and four). The dependant variable was the number of episodes (self-harm or violent). The independent variables were time (a continuous variable representing time in months since November 1st 2016), a binary variable indicating period before or after the intervention and an interaction term between time and intervention. We conducted a sensitivity analyses using a restricted follow-up of 12 months so that the majority of prisoners could be included. All analyses were performed using IBM SPSS Version 26 [44].

Segmented regression analysis of ITS data assesses how much an intervention changed an outcome, immediately and over time. A sufficient number of time points is required before and after the intervention in order to conduct a segmented regression analysis, a general recommendation is 12: our analyses exceed the number of recommended time points both before and after the intervention [19]. One of the problems with the ITS analysis is autocorrelation where error terms of consecutive observation are correlated: if present and left uncorrected it can lead to underestimates of the standard errors and an over-estimate of the effect of the intervention. The Durbin- Watson statistic was used to check for autocorrelation, the values were all within the accepted range (close to a value of 2) which ruled out any significant effects of auto-correlation [45].

### 4.3 Qualitative data analyses

Qualitative data were transcribed anonymously, transferred into different themes and then data uploaded using the qualitative software N-Vivo [46]. A thematic analysis of the content was applied using a framework [47].

### 4.4 Follow-up study analyses

Parametric and non-parametric tests were applied using SPSS software to assess the distribution of the data. Significance testing at *p*<0.05 assessed for differences between the group on baseline demographic characteristics (age, length of sentence, attendance at a previous training course or offence type) and at follow-up.

### 4.5. Role of the funding source

The funders reviewed the study proposals, awarded the funding and monitored the conduct of the study. The funders had no role to play in the study design, data collection, data analysis, data interpretation or writing of the report. The corresponding author had full access to all data in the study and had final responsibility for the decision to submit for publication.

## 5 Results

### 5.1 Stage one: the theory of change causal map

**Fig. 3:**
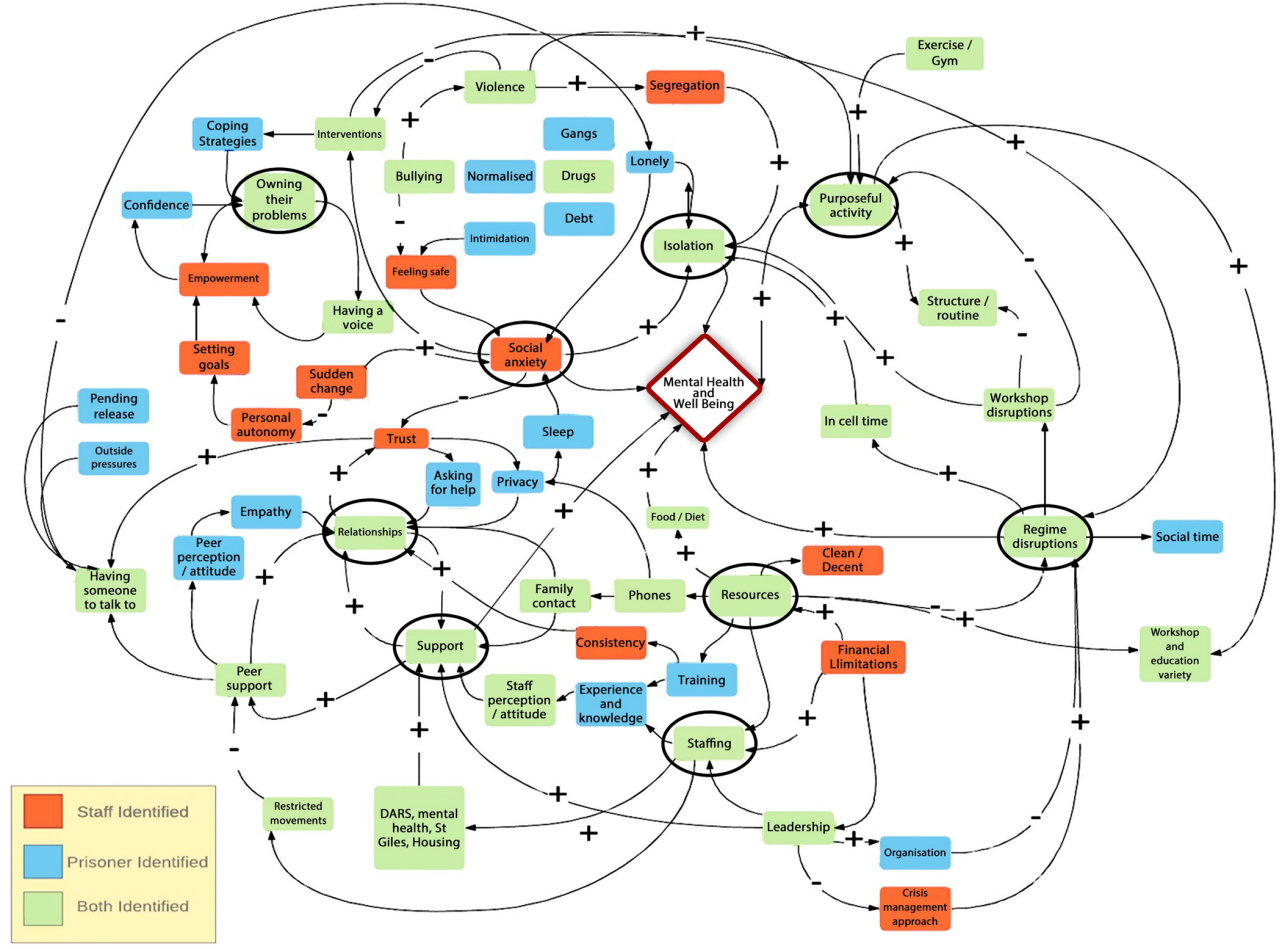
A causal map on the factors impacting on mental health and well-being in prison.

20 participants (10 staff and 10 PSMs) attended three separate workshops. Prison staff represented various stakeholders to enhance ‘buy in’ and support a collaborative approach to the eventual implementation of the intervention. The causal map (Fig. 3) represents the responses generated by prison staff and PSMs. Nine higher level constructs included support, staffing, resources, regime disruptions, purposeful activity, isolation, social anxiety, owning their own problems and relationships (supplementary Appendix D). To support the nine higher level constructs the training manual was adapted to incorporate specific group exercises and prompted facilitator questions were added to each session.

### 5.2 Stage two: implementation of the peer-led problem support mentor intervention

Between 26th, February, and 13th, December 2018, 36 prisoners representing 11/14 wings were trained to become a PSM in four successive groups (Table 1).

**Table 1:** Characteristics of the problem support mentors.

Demographic characteristic (Total *N* = 36)	Category	
Age (*N* = 32)	*Mean* (SD) [min, max]	*33.25* (10.32) [22,65]
Demographic characteristic (Total *N* = 36)	Category	*N* (%)
Ethnic Background (*N* = 33)	British	21 (64)
	White and Black Caribbean	4 (12)
	White and Asian	1 (3)
	Indian	1 (3)
	Pakistani	2 (6)
	Caribbean	4 (12)
Religious Background (*N* = 32)	No Religion	13 (41)
	Muslim	5 (16)
	Sikh	1 (3)
	Buddhist	2 (6)
	Christian	11 (34)
Marital status (*N* = 33)	Married/partnership	10 (30)
	Divorced/separated	1 (3)
	Single - never married	22 (67)
Academic attainment (*N* = 31)	Postgraduate/NVQ5	2 (6)
	Degree level	6 (19)
	A level/BTEC/City and Guilds	15 (48)
	GCSE/CSE/NVQ1	7 (23)
	None	1 (3)
First time offender (*n* = 33)	No	23 (70)
	Yes	10 (30)
No. of times in prison (*n* = 30)	1	10 (33)
	2–4	12 (40)
	5 or more	8 (27
Length of time in this prison (*N* = 33)	<=6 months	13 (39)
	7 – 12 months	10 (30)
	12 −24 months	8 (24)
	>24	2 (6)
Sentence length (*N* = 32)	<=24 months	2 (6)
	>24 – 48 months	11 (34)
	>48 months – 72 months	9 (28)
	>72 months	10 (31)
Type of offence (*N* = 31)	Robbery	5 (16)
	Fraud	4 (13)
	Conspiracy To Supply Class A	4 (13)
	Possession Intent To Supply^a^	4 (13)
	Murder	2 (6)
	Section 20 (GBH)^b^	2 (6)
	Dangerous Driving	1 (3)
	Section 18 (Wounding)	4 (13)
	Possession Class B	1(3)
	Money Laundering	1 (3)
	Burglary	2 (6)
	Assault	1 (3)

a Possession with intent to supply – refers to supplying or offering to supply a controlled drug/ possession of a controlled drug with intent to supply it to another.

b Section 20 (GBH) – refers to causing grievous bodily harm injuries without the intention to cause such severe harm.

Their mean age was 33 years (SD 10.32) with the majority of white ethnicity 21/36 (58%), most were single and had never been married 22/36 (61%). Over half, 20/36 (55%) had been in prison four or more times. When starting the intervention, 23/36 (64%) had been in the prison 12 months or less. Thirty-one (86%) reported a range of offence details. Most, 31/36 (83%) had a sentence for 24 months or more. On starting the intervention living conditions based on the prison service incentive policy framework scheme (IPF) reported 9/36 (25%) on ‘enhanced’ and 9/36(25%) on ‘standard’ level (missing data were recorded for the remainder of the sample). More than half 19/36 (52.7%) had previously completed an accredited prison program.

### 5.3 Adherence to the PSM training

Training was conducted at four successive time periods. Group one; from 27.2.2018 (11 people), group two from 6.3.2018, (seven people), group three from 13.7.18 (eight people) and group four from 13.12.18 (ten people). Two of the 36 (5.5%) chose to be part of the intervention for less than one month. The majority 22/36 (61%) remained in the intervention for four months or more and a quarter 9/22 (25%) engaged for more than 36 weeks (range 2 to 48 weeks,). Two PSMs were removed from the intervention by prison staff due to behaviour that was not thought to be representative of the intervention. Five of the 36 (13.8%) disengaged during the intervention. Reasons for disengagement included: bereavement, feeling uncomfortable with the training and prioritisation of other prison jobs. During the intervention, 8/36 (22%) people moved wings once, and one person moved wings twice (supplementary file Appendix E).

### 5.4 Adherence to the PSM intervention delivery

Between March 1st and June, 14th 2019, 828 peers received one or other variant of the intervention: 698/828 (84%) received promotion only and 130/828 (16%) received the full PST skills. 249/828 (30%) had a history or current incidence of violence and 130/828 (16%) had a history or current incident of self-harm.

Of those receiving promotion only, 425/698 had no self-harm or violence record, 141/698 (20%) had a violent incident and 111/698 (16%) an incident of self-harm. 21/698 (3%) prisoners had a history of both self-harm and violence. Those receiving the full PST skills included 108/130 had a violent incident, 19/130 had an incident of self-harm and three prisoners had a history of both self-harm and violence. The average length of the intervention delivery for the full skills was 18 min (range 3–120 min), and for promotion of the intervention was 3 min (range 2–10 min).

The majority of referrals came through the induction wing where most prisoners were provided with promotion of the intervention (89%). Staff referrals made up a small proportion of other referrals 42/828 (5%) and a minority of PSMs approached other peers 35/828 (4%).

Of those receiving the full PST skills PSMs recorded the detail of the problem in 76/130 (58%) cases. Problems included drugs and thoughts of self-harm, 15/76 (19.7%), bullying and violence 5/76 (6.5%), adjustment to prison life, 7/76 (9.2%), contacting family and social networks, 9/76 (11.8%), problems relating to release, (including employment, contact with probation, rules around the electronic tag system and issues relating to housing) 20/76 (26.3%), access to healthcare, 2/76 (2.6%), wanting to speak to someone, 2/76 (2.6%), organisational logistics (including complaints, problems with lost property and clothing, access to writing materials and wanting employment within the prison)11/76 (14.4%) and debt 5/76 (6.5%) (supplementary file Appendix F).

PSMs’ actions to support peers occurred in 59/130 (45%) sessions where skills were delivered. Actions included explaining how the prison system worked and supporting access 30/59 (50.8%), aiding communication between staff and prisoners 6/59 (10.2%), talking 3/59 (5.1%), use of the PST skills to look at all available options and devising a plan 16/59 (27.1%), provision of emotional support and encouragement 3/59 (5.1%) and workbook materials 1/59 (1.7%).

### 5.5 ITS results: self-harm outcomes for the total prison population (group one)

**Fig. 4:**
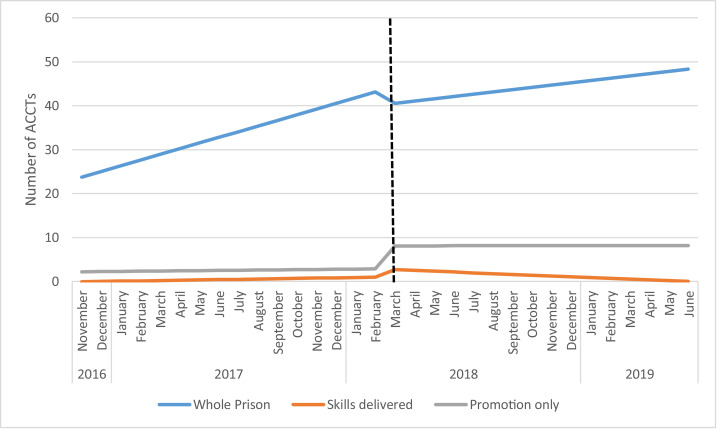
Incidence ACCTs November 2016 to June 2019 (Estimates from ITS analyses).

Between November, 1st 2016 and June, 14th 2019, the prison recorded a total of 1246 self-harm incidents (Fig. 4). The average number of incidents in the 16 months before the intervention began was 35 per month this ranged between 27 and 43. During and post intervention this increased over the following 15 month period to an average of 44 per month, ranging between and 28 and 60.

The first analysis examined the total number of ACCTs for the whole prison. Over time the number of incidents was increasing, (1.29, 95%CI 0.34, 2.24) this was significant (Table 2). After the introduction of the intervention the number of incidents dropped (−3.11, 95%CI −15.58, 9.36) but not significantly and there was no significant change after the intervention (0.77, 95%CI −2.11, 0.58).

**Table 2:** ACCT incidences and ITS analyses.

	Whole Prison	Skills delivered	Promotion only
	Estimate	Estimate	Estimate
	(95% CI)	(95% CI)	(95% CI)
	*p*-value	*p*-value	*p*-value
Intercept (β_0_)	22.48	−0.15	2.125
	(13.26, 31.69)	(−0.978, 0.678)	(−1.478, 5.728)
	*P*<0.001	*P* = 0.713	*P* = 0.237
Baseline Trend (β_1_)	1.29	0.07	0.04
	(0.34, 2.24)	(−0.02, 0.16)	(−0.41, 0.56)
	*P* = 0.010	*P* = 0.109	*P* = 0.761
Level change after intervention (β_2_)	−3.11	1.89	5.2
	(−15.58, 9.36)	(0.78, 3.01)	(0.37, 10.12)
	*P* = 0.613	*P* = 0.002	*P* = 0.036
Trend change after intervention (β_3_)	−0.768	−0.25	−0.04
	(−2.11, 0.58)	(−0.37, −0.13)	(−0.57, 10.12)
	*P* = 0.253	*P*<0.001	*P* = 0.883

Regression Model.

*Y* = β_0_ + β_1_*T1 +β_2_*intervention + β_3_*T2 +ε.

*Y* = number of ACCTs or violent episodes each month.

T1 = Time from the start of the observation period.

T2 = Time since the start of the intervention.

### 5.6 ITS results: self-harm outcomes in those receiving the intervention (group two)

The ITS analysis for the full PST skills delivered group found no significant increase in the number of at risk incidents over time (0.07, 95%CI −0.98, 0.68). After the introduction of the scheme there was a significant increase in reporting of 1.89 per month ACCTs (95% CI 0.78, 3.01). The change after the intervention was 0.25 per month (95%CI −0.37, −0.13).

The ITS analysis for the promotion only group found no significant increase in the number of at risk incidents over time (0.04, 95%CI −0.41, 0.56), a significant increase after the intervention (5.2, 95%CI 0.37, 10.12) but no evidence that the rate changed after the introduction of the intervention (−0.04, 95% −0.57, 10.12). A sensitivity analysis was conducted using a shorter time period so that all those prisoners would be included. These analyses confirmed the results, indicating that the results were not affected by the release/transfer of prisoners before the end of the analysis period.

### 5.7 ITS results: violence outcomes for the total prison population (group three)

**Fig. 5:**
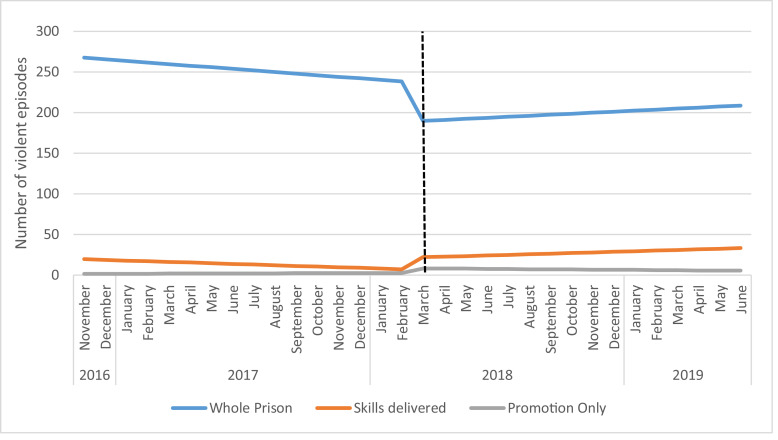
Incidence of violence November 2016 to June 2019 (Estimates from ITS analyses).

Between November, 1st 2016, and June 2019, the prison recorded a total of 7234 violent incidents (Fig. 5). The average number of incidents in the 16 months before the intervention began was 256 per month. During and post intervention this decreased over the following 15 month period to an average of 203 per month.

Over time the number of violent episodes for the whole prison was not significantly changing (−1.95, 95%CI −8.95, 5.05). There was no significant change in the number directly after the intervention (−49.9, 95% CI −141.39, 41.59) and no change in the trend (3.22, 95%CI-6.68, 41.59). Similar results were seen in the skills delivered group, at baseline (−0.82, 95%CI −2.6,0.96), level change (140.03, 95%CI −9.22, 37.27) and trend change (1.58, 95%CI −0.95, 4.08). This was repeated in the promotion only group where the baseline was 0.08 (95%CI −0.41, 0.57) level change 5.70 (95%CI −0.67, 12.07) and trend change was −0.27 95%CI (−0.96, 0.42) (Table 3).

**Table 3:** Violent incidents and ITS analyses.

	Whole Prison	Skills delivered	Promotion only
	Estimate	Estimate	Estimate
	(95% CI)	(95% CI)	(95% CI)
	*p*-value	*p*-value	*p*-value

Intercept (β_0_)	269.50	20.43	1.53
	(201.85, 337.15)	(3.24, 37.61)	(−3.18, 6.23)
	*P*<0.001	*P* = 0.022	*P* = 0.513
Baseline Trend (β_1_)	−1.95	−0.82	0.08
	(−8.95, 5.05)	(−2.60, 0.96)	(−0.41, 0.57)
	*P* = 0.573	*P* = 0.671	*P* = 0.745
Level change after intervention (β_2_)	−49.90	14.03	5.70
	(−141.39, 41.59)	(−9.22, 37.27)	(−0.67, 12.07)
	*P* = 0.273	*P* = 0.227	*P* = 0.077
Trend change after intervention (β_3_)	3.22	1.57	−0.27
	(−6.68, 41.59)	(−0.95, 4.08)	(−0.96, 0.42)
	*P* = 0.511	*P* = 0.212	*P* = 0.435

**Regression Model**.

*Y* = β_0_ + β_1_*T1 +β_2_*intervention + β_3_*T2 +ε.

*Y* = number of ACCTs or violent episodes each month.

T1 = Time from the start of the observation period.

T2 = Time since the start of the intervention.

### 5.8 ITS results: violence outcomes in those receiving the intervention (group four)

During the intervention 249 people with a current or historical incident of violence were seen by a PSM; 108/249 (43%) received the full PST skills and 141/249 (56%) received promotion only. 14/249 (5%) were seen on a second occasion by the PSM, and 2/249 (0.8%) were seen on a third occasion. Of the 249, 104 (42%) were reported to have a history of violence before the intervention began representing 369/4047 (9%) of the total number of violent incidents in the prison.

### 5.9 Qualitative results

13/36 (36%) PSMs were interviewed. Three higher level thematic categories were identified, (i) selection and training, (ii) development of self-confidence and (iii) development of personal skills (iv) operational support from the wider prison. Within these categories several subordinate themes were identified (supplementary file Appendix H).

### 5.10 Theme one: selection and training

The delivery of the peer led intervention was dependant on the selection and recruitment of effective PSMs (Table 4). Successful candidates benefited from characteristics and qualities including; confidence, maturity, being assertive, trustworthy, empathetic, positive and with good communication/active listening skills. The majority felt it important to use prison-based training examples to support them in their roles. The sustainability of the intervention included suggestions for co-facilitation and support for an outside organisational partnership.

**Table 4:** Qualitative examples for theme one selection and training.

Theme one: selection and training
*“I think it needs to be somebody that stands out positive that can speak, somebody that can interact with others”* *“someone needs to approachable and not scared to voice their opinions where it needs to be voiced”,* *“the last thing that they want is you're not to be fully there, not to listen, not to engage, not to interact, not to have eye contact”* *“as long as people have got the knowledge and people are doing the training, obviously people will be getting out, people will be going to cat D, people will get HDC. “*

### 5.11 Theme two: development of self-confidence

PSMs reported benefits to their own development and progression. Examples included helping others, providing a sense of purpose and fulfilment, improved relationships with prison staff and improved communication skills (Table 5).

**Table 5:** Qualitative examples for theme two development and confidence.

Theme two: development and confidence
“*Well no because I like it, to be honest with you. It's what changed me, is helping other people, this time round”* *“I think it has benefited me, because it's taken me out of my comfort zone”* *“I've been able to liaise with people that I wouldn't really liaise with or talk to or officers”* *“I must say it gave me a lot of confidence”* *“when I was not here on this course before my thinking was different, and when I got these courses, after a couple of lessons, the confidence came in”.*

### 5.12 Theme three: development of personal skills

The intervention supported changes in cognitive thinking (Table 6). Some PSMs reported that completing the process of the seven steps helped them to ‘*slow down’*. Others commented on how relatively minor problems could be addressed to alleviate stressful symptoms and empower other peers to solve their own problems.

**Table 6:** Qualitative examples for theme three development of personal skills.

Theme three: development of personal skills
*“The officer referred him to me and he says, have you heard about seven steps and then the guy come to my pad, a bit of a mess anyway, you know what I mean, so I sat him down and talked to him, gave him a cup of coffee and stuff like that. I calmed him down and said, it's not as bad, because it were his first time in jail and he kept doing his wrists and stuff.* *“Yes, I went in my pad, I knew I was angry, I wanted to do something, I'd rather smash my pad up or go and see the kid……I wasn't thinking about that when I was in rage but (the mentor) obviously sat me down and brought it back into me, he planted it back into me that you need to stop, think about this sort of stuff instead of getting yourself into trouble”.* *“When I went to (mentor), I was up in a raw, I wanted to go and hurt someone, before I went to go and see the officer, he (mentor) sat me down and talked to me, calmed me down, do you know what I mean?* *“that's what makes it a good thing because you're not telling them, do this, do that, because it might not work for them what…how you've solved yours, but, for example, you brainstorm, or pros and cons”* *“it takes a lot of stress off of staff with just minor issues that we can help them out with and stuff like that and then how they should go about the problem themselves*” “*it's somebody on the ground level before anything escalates and it's goes further and further and further, they can actually deal with the issue. Things like this could be nipped in the bud)”*

### 5.13 Theme four: operational support from the wider prison

Staff engagement and organisational support was key to the scheme (Table 7). 22% of PSMs at time 1 (May 2018) reported feeling supported and utilised by staff in the PSM role. As the scheme became more embedded within the prison this increased to 75% at time 2 (October 2018).

**Table 7:** Qualitative experiences theme four operational support from the wider prison.

Theme four: operational support from the wider prison
*“they're just pushing it under the carpet; you tell them, oh, I've got someone to see you for problem-solving; and they just say, oh, yeah, whatever)”.* *It's growing. They're more aware of it. Obviously, like I say, if you have a permanent up on the wing with a box then the staff are more aware of it, you know what I mean, because some are starting to refer. So, it is climbing, you know?.* “ *it's alright the inmates thinking it's a good idea but if it's not supported by people up at the top, then it's never going to happen.”* *“you see because they resort to self-harm, they resort to drugs, they resort to things like that to sort their problems out, when it should say at the front, look as soon as they get put on an ACCT, if they want to, we can refer them to this lad here to go and have a word with him, because a lot of people will listen to their peers more, rather than the staff, don't they?”.*

### 5.14 Stage three: follow up study results

At baseline no significant differences were revealed between controls and cases for age, length of sentence, attendance at a previous training course or offence type (supplementary file Appendix G). Controls were more likely to be living on ‘basic’ (4 v 0) or ‘standard’ (12 vs. 7) living conditions and less likely to be living on ‘enhanced’ (13 vs. 23) in comparison to cases (*p* = 0.014). At follow-up 35/72 (48%) were released, 26/35 (74%) of those released remained in the community whilst 9/26 (34%) returned to custody. 37/72 (51%) remained in custody. Of those who remained in custody 17/37 (45%) were transferred to another prison (Table 8). No differences between controls and PSMs (cases) were found in rates of return to prison although the numbers in the sample were small. 100% follow-up data was obtained, and excluded five people with a sentence of life and two people with an indeterminate sentence

**Table 8:** Follow-up on reoffending rates up to 16 months.

Cohort group	Released (%)	Remains in custody (%)	Remains in custody but transferred to another prison (%)	Following release remains in the community (%)	Following release returned or recalled to custody (%)
Cases *n*	18/36 (50)	19/36 (52)	9/19 (50)	13/18 (72)	4/18 (22)
Controls *n*	17/36(47)	18/36 (52)	9/18 (50)	13/17 (76)	5/17 (29)
Total group *n*	35/72 (48)	37/72 (51)	18/37 (48)	26/35 (74)	9/26 (34)

## 6 Discussion

Using co-production we adapted an existing PST skills intervention to produce a ToC model. The ToC model supported the idea that the mental health and well-being of prisoners was affected by environmental, social and cultural aspects of the prison. National and international policy on the management of self-harm and violent behaviour should consider how the design and implementation of future interventions should take into account a holistic approach to supporting the prevention of self-harm and violent behaviour [48,49].

Four successive groups of PSMs were trained in the 10-month period and only a small minority of participants disengaged early. Qualitative findings suggested that identification of the ‘right type of person’ to be a mentor was key to the successful running of the intervention. PSMs although carefully selected still experienced problems of their own. We did not collect data on those people that were screened for the role and were not chosen. Such data may inform the identification of a minimum data set for future mentors. Additional prison sites and populations (e.g., females and juveniles) are required to explore the generalisability of these results.

Most problems related to difficulties in arranging housing, employment and benefits upon release and logistical problems about how the prison system worked. Logistical problems were indicative examples of how relatively small problems can escalate. Local strategies could be improved to provide a consistent and comprehensive induction program which would better manage the expectations for people on arrival at prison. Future interventions could target prisoners upon entry and within one month prior to release. Other studies have shown these periods of entry and release can exacerbate mental health problems [50].

The intervention received relatively few referrals from prison staff. Anecdotal evidence suggested that staff were concerned about risk sharing and information; however during the intervention we had no adverse events to report. Culturally, prisons need to utilise the skills of those incarcerated empowering individuals to cope and address problems in a proactive, considered manner.

We used an ITS analyses to evaluate the impact of a brief peer-led PSM intervention on self-harm and violent behaviour using routinely collected data. Despite the brief nature of the intervention and the small numbers of those involved in the intervention delivery we did find a significant reduction in the incidence of self-harm for those who received full delivery of the PST skills. There was no evidence that the brief intervention led to a significant reduction in people at risk of self-harm or violent episodes for the whole prison. Limitations of the ITS design meant that routinely collected data were susceptible to other changes in the prison environment, such as tightened regime restrictions or a prisoner suicide. Incidences of violent behaviour were much higher than self-harm and included an eclectic mix of behaviour, some more akin to ‘rule breaking’ than violence. Qualitative findings supported individual personal development goals of those taking part in the scheme and encouraged the holistic buy-in required to support the implementation of the intervention by staff. Follow-up in the community showed the feasibility of collecting such data for a definitive study.

The multi-levelled holistic approach to intervention implementation and efficient model that utilised existing routinely collected data enabled us to ascertain the impact of the intervention at a number of different levels. As a small scale study the results show promise, expansion of the scheme into other prison sites and access to measures of health diagnoses, measures of reliable clinical change and quality of life and economic costings would be imperative in a large scale study of effectiveness. Future implementation of mental health interventions should consider a comprehensive multi-agency collaboration [9].

## Data sharing

Additional materials may be requested after approval from the corresponding author and HMPPS.

## Funding

This work was supported by the Research Champions fund and the Economic and Social Research Council Impact Acceleration Fund, University of York, UK.

## Contributors

AP, AH and MGW were responsible for the overall design of the study. AP oversaw the day to day conduct of the study. VD conducted the statistical analysis. KM and AP supported the workshops and the qualitative analyses of the mentors and customers, KS conducted the qualitative analyses for the staff members. All authors made substantial contributions to the interpretation of the data and drafting of the article.

## Declaration of Interests

All authors declare no competing interests.

## Appendix Supplementary materials

Image, application 1
