# Supplementary material for: The effect of a peer-led problem-support mentor intervention on self-harm and violence in prison: An interrupted time series analysis using routinely collected prison data
Source: eClinicalMedicine. 2021 Jan 15;32:100702. doi: 10.1016/j.eclinm.2020.100702 (PMC7910675; doi:10.1016/j.eclinm.2020.100702)
Supplement: Supplementary file 1 [file mmc1.docx]

**Web Appendix Supplementary file information**

Appendix A: Supplementary meta-analyses of problem-solving interventions and references

Appendix B: Theory of Change Model

Appendix C: Table one violent behaviours

Appendix D: Table two barriers and facilitators to the theory of change model

Appendix E: Table three change in wing by problem support mentor

Appendix F: Figure 5 Problems dealt with by problem support mentors

Appendix G: Table four case and controls baseline characteristics

Appendix H: Table five qualitative themes

Appendix A: Supplementary meta-analyses of problem solving interventions

Notes on the systematic review methodology:

Studies with interventions of PST and other components were excluded ^43,44^. Studies with overlapping adolescent *and* adult populations were included. All studies required an outcome of either repetition of self-harm or a measure of suicidal ideation. Studies were divided into groups based on the intervention comparison and follow-up time periods.

Figure1: Problem solving therapy versus treatment as usual for depression, suicidal ideation and hopelessness at up to four months


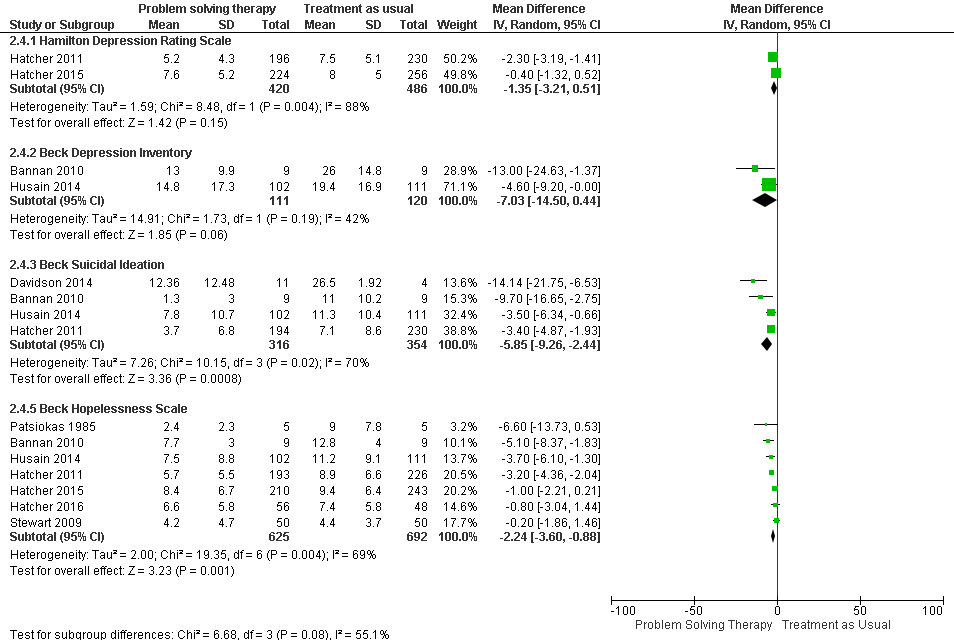


Figure 2: Problem solving therapy versus waiting list control for depression, suicidal ideation and hopelessness at up to four months follow up


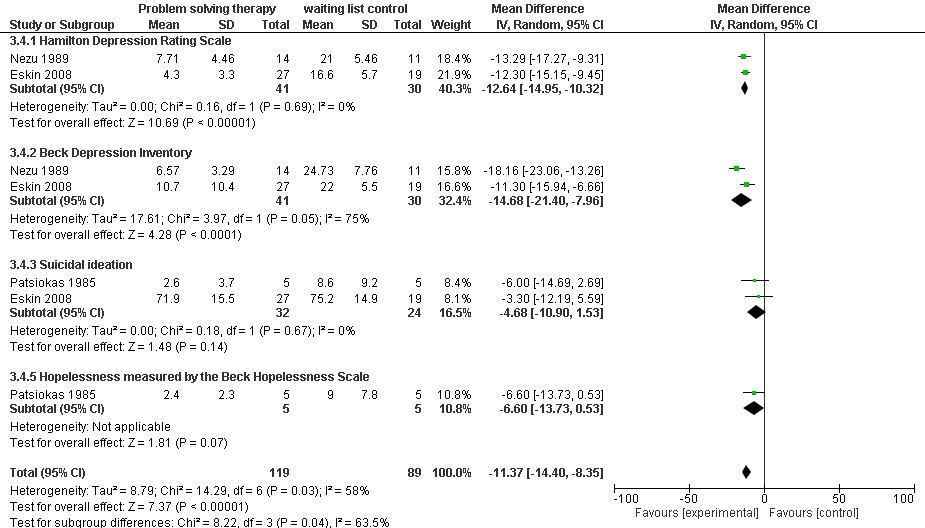


Figure 3: Problem solving therapy versus alternative or brief therapy for depression, suicidal ideation and hopelessness at up to 4 months


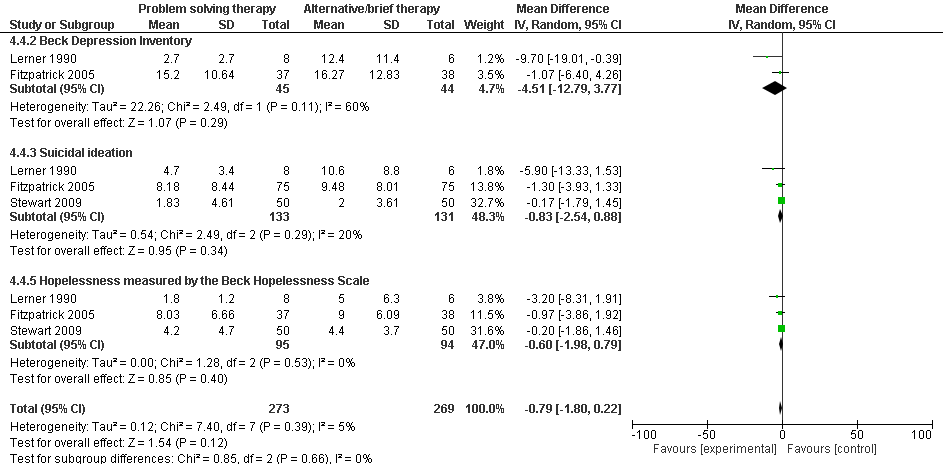


**Included studies * in the meta-analyses**

1. Bannan N. Group-based problem-solving therapy in self-poisoning females: A pilot study. *Counselling and Psychotherapy Research* 2010; **10**(3): 201-13.*

2 McAuliffe C, McLeavey BC, Fitzgerald T, et al. Group problem-solving skills training for self-harm: randomised controlled trial. *The British Journal of Psychiatry* 2014; **204**: 383–90. doi: 10.1192/bjp.bp.111.101816.*

3. Husain N, Afsar S, Ara J, et al. Brief psychological intervention after self-harm: randomised controlled trial from Pakistan. *British Journal of Psychiatry* 2014; **204**: 462-70.*

4. Xavier A, Otero P, Blanco V, Vazquez FL. Efficacy of a problem-solving intervention for the indicated prevention of suicidal risk in young Brazilians: Randomized controlled trial. *Suicide and Life Threatening Behaviour* 2019; **49**: 1746-61.*

5. Evans K, Tyrer P, Catalan J, et al. Manual Assisted Cognitive Behavioural Therapy in the Treatment of Recurrent Deliberate Self Harm: A Randomised Controlled Trial. . *Psychological Medicine* 1999; **29**: 19-25.*

6. Gibbons JS, Butler J, Urwin P, Gibbons JL. Evaluation of a social work service for self-poisoning patients. *The British journal of psychiatry : the journal of mental science* 1978; **133**(2): 111-8.*

7. Hawton K, Bancroft J, Catalan J, Kingston B, Stedeford A, Welch N. Domiciliary and out-patient treatme of self-poisoning patients by medical and non-medical staff. *Psychological Medicine* 1981; (11): 169-77.*

8. Hawton K, McKeown S, Day A, Martin P, O'Connor M, Yule J. Evaluation of out-patient counselling compared with general practitioner care following overdoses. *Psychol Med* 1987; **17**(3): 751-61.*

9. Hatcher S, Coupe N, Wikiriwhi K, Durie M, Pillai A. Te Ira Tangata: a Zelen randomised controlled trial of a culturally informed treatment compared to treatment as usual in Ma¯ori who present to hospital after self-harm. *Soc Psychiatry Psychiatr Epidemiol* 2016; **51**: 885–94.*

10. Hatcher S, Sharon C, House A, Collins N, Collings S, Pillai A. The ACCESS study: Zelen randomised controlled trial of a package of care for people presenting to hospital after self-harm. *The British Journal of Psychiatry* 2015; **206**: 229-36 doi: 10.1192/bjp.bp.113.135780.*

11. Hatcher S, Sharon C, Parag V, Collins N. Problem-solving therapy for people who present to hospital with self-harm: Zelen randomised controlled trial. *The British journal of psychiatry : the journal of mental science* 2011; **199**(4): 310-6.*

12. Salkovskis PM, Atha C, Storer D. Cognitive-Behavioural Problem Solving in the Treatment of Patients who Repeatedly Attempt Suicide: A Controlled Trial. *British Journal of Psychiatry* 1990; **157**: 871-6.*

13. Davidson KM, Brown TM, James V, Kirk J, Richardson J. Manual-assisted cognitive therapy for self-harm in personality disorder and substance misuse:a feasibility trial *Psychiatric Bulletin* 2014; **38**: 108-11 doi: 10.1192/pb.bp.113.043109.

14. Ramani Perera EA, Kathriarachchi ST. Problem—solving counseling as a therapeutic tool on youth suicidal behavior in the suburban population in Sri Lanka. *Indian J Psychiatry* 2011; **Jan-Mar; 53(1)**: 30-5 doi: 10.4103/0019-5545.75558:.

15. Slee N, Garnefski N, van der Leeden R, Arensman E, Spinhoven P. Cognitive-behavioural intervention for self-harm: randomised controlled trial. *The British journal of psychiatry : the journal of mental science* 2008; **192**(3): 202-11.*

16. Eskin M, Ertekin K, Demir H. Efficacy of a problem-solving therapy for depression and suicide potential in adolescents and young adults. *Cognitive Ther Res* 2008; **32**: 227-45*

17. Nezu AM. Efficacy of a social problem-solving therapy approach for unipolar depression. *Journal of Consulting and Clinical Psychology,* 1986; **54**: 196-202.

18. Patsiokas AT, Clum GA. Effects of psychotherapuetic strategies in the treatment of suicide attempters. *Psychotherapy* 1985; **22**.*

19. Lerner MS, Clum GA. Treatment of Suicide Ideators: A Problem-Solving Approach *Behaviour therapy* 1990; **21**: 403-11.*

20. Parker AG, Hetrick SG, Jorn AF, et al. The effectiveness of simple psychological and physicalactivity interventions for high prevalence mental health problems in young people: A factorial randomised controlled trial. *Journal of Affective Disorders* 2016; **196**: 200-9.*

21. Stewart CD, Quinn A, Plever S, Emmerson B. Comparing Cognitive Behavior Therapy, Problem Solving Therapy, and Treatment as Usual in a High Risk Population. *Suicide and Life-Threatening Behavior* 2009; **39**(5): 538-46.*

22. Fitzpatrick KK, Witte TC, Schmidt NB. Randomized Controlled Trial of a Brief Problem-Orientation Intervention for Suicidal Ideation. *Behavior Therapy* 2005; **364**: 323-33.*

23. Gustavson KA, Alexopoulos GS, Niu G, McCulloch G, Meade T, Arean PA. Problem-Solving Therapy Reduces Suicidal Ideation In Depressed Older Adults with Executive Dysfunction. *Am J Geriatr Psychiatry* 2016; (1): 11–7. doi:0.1016/j.jagp.2015.07.010

**Excluded studies at second stage screening**

1. Donaldson D, Spirito A, Esposito-smythers C. Treatment for Adolescents Following a Suicide Attempt: Results of a Pilot Trial. *J AM Acad Child Adolesc Psychiatry* 2005; **44**(2): 113-20.

2. Batterham PJ, Caleara AL, Farrera L, McCalluma SM, Wan Sze Cheng V. FitMindKit: Randomised controlled trial of an automatically tailored online program for mood, anxiety, substance use and suicidality. *Internet Interventions* 2018; **12**: 91-9.

3. Black DW, Allen J, St. John D, Pfoh lB, B. M, Blum N. Predictors of response to systems training for emotional predictability and problem solving (STEPPS) for borderline personality disorder: an exploratory study. *Acta Psychiatr Scand* 2009; **120**: 53-61 DOI: 10.1111/j.600-0447.2008.01340.x.

4. Brown GK, Ten Have T, Henriques GR, Xie SX, Hollander JE, BecK AT. Cognitive therapy for the prevention of suicide attempts: a randomized controlled trial. . *Journal of the American Medical Association* 2005; **294**(5): 563-70.

5. Tyrer P, Thompson S, Schmidt U, et al. Randomized controlled trial of brief cognitive behaviour therapy versus treatment as usual in recurrent deliberate self-harm: The POPMACT study. *Psychological Medicine* 2003; **6**: 969-76.

6. Blum N, St. John D, Pfohl B, et al. Systems Training for Emotional Predictability and Problem Solving (STEPPS) for Outpatients With Borderline Personality Disorder: A Randomized Controlled Trial and 1-Year Follow-Up. *Am J Psychiatry* 2008; **165**: 468-78 doi:10.1176/appi.ajp.2007.07071079.

7. De Jaegerea E, van Landschoot R, van Heeringen K, et al. The online treatment of suicidal ideation: A randomised controlled trial of an unguided web-based intervention. *Behaviour Research and Therapy* 2019; **119**.

8. Morthorst B, Krogh J, Erlangsen A, Alberdi F, Nordentoft M. Effect of assertive outreach after suicide attempt in the AID (assertive intervention for deliberate self harm) trial: randomised controlled trial. *Bmj* 2012; **345**: e4972.

9. Choudhury N, Hicks RC, Kreitman N. Evaluation of an after-care service for parasuicide (attempted suicide) patients. *Social Psychiatry* 1973; **8**: 67-81.

10. Miller IW, Carlos A, Camargo J, et al. Suicide Prevention in an Emergency Department Population The ED-SAFE Study. *JAMA Psychiatry* 2017; **74**(6): 563-70.

11. Riaz R, Agha S. Efficacy of Cognitive Behavior Therapy with Deliberate Self-harm in Incarcerated Women. *Pakistan Journal of Psychological Research* 2012; **27**(1): 21-35.

12. van Spijker BAJ, van Straten A, Kerkhof JFM. Effectiveness of Online Self-Help for Suicidal Thoughts: Results of a Randomised Controlled Trial. *PloS one* 2014; (2): e90118.

13. Atha C, Salkovskis PM, Storer D. Cognitive behavioural problem solving in the treatment of patients attending a medical emergency department: A controlled trial. *Journal of Psychosomatic Research* 1992; **36**(4): 299-307.

Appendix B: Theory of Change Model


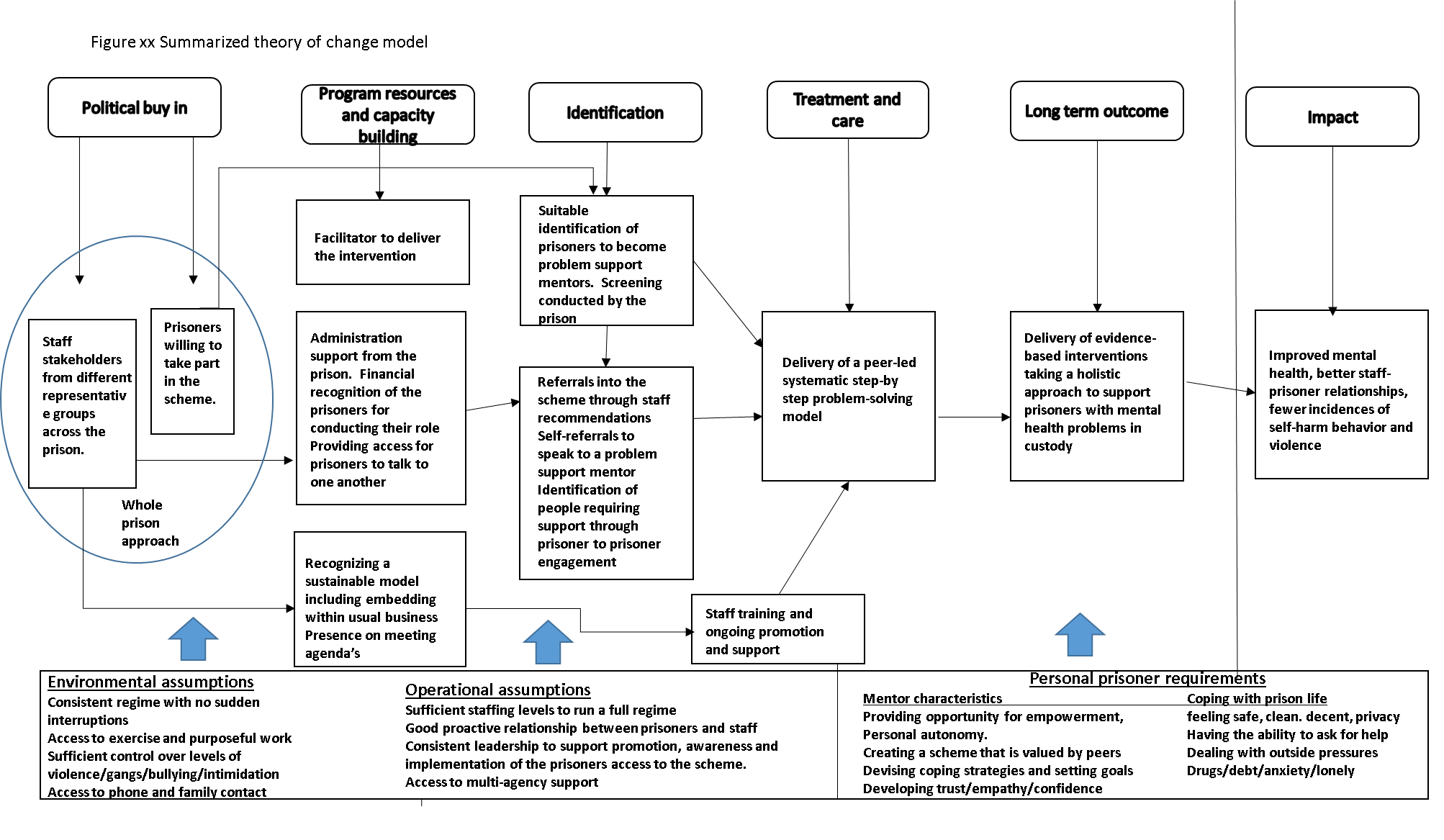


Appendix C: Table one violent behaviours

| Reported behaviour | Staff identification number () and ranking^[[1]](#footnote-1)^ | | | | | | | | | | | | | | Overall mean ranked number | | Behaviour ranked in order^[[2]](#footnote-2)^ | |
| --- | --- | --- | --- | --- | --- | --- | --- | --- | --- | --- | --- | --- | --- | --- | --- | --- | --- | --- |
|  | (1) | (2) | (3) | (4) | (5) | (6) | (7) | (8) | (9) | | (10) | | (11) |  | | | |  |
| assault | 17 | 16 | 17 | 17 | 17 | 16 | 14 | 17 | | 17 | | 17 | 17 | 16.5 | | 17 | |  |
| fight | 15 | 13 | 15 | 12 | 16 | 15 | 14 | 16 | | 16 | | 11 | 17 | 14.5 | | 15 | |  |
| fire | 18 | 4 | 16 | 16 | 18 | 17 | 14 | 18 | | 11 | | 13 | 16 | 14.6 | | 16 | |  |
| threats | 13 | 10 | 13 | 11 | 12 | 14 | 11 | 15 | | 14 | | 4 | 12 | 11.7 | | 14 | |  |
| endangers | 16 | 3 | 10 | 14 | 15 | 4 | 13 | 13 | | 9 | | 18 | 15 | 11.8 | | 13 | |  |
| barricade | 10 | 5 | 12 | 15 | 11 | 13 | 12 | 9 | | 10 | | 14 | 13 | 11.3 | | 12 | |  |
| self-harm | 4 | 18 | 4 | 13 | 13 | 7 | 9 | 11 | | 13 | | 16 | 9 | 10.6 | | 11 | |  |
| drugs | 9 | 17 | 10 | 9 | 7 | 12 | 10 | 5 | | 10 | | 5 | 11 | 9.5 | | 9 | |  |
| Under the influence | 12 | 9 | 8 | 7 | 7 | 6 | 9 | 14 | | 15 | | 12 | 7 | 9.6 | | 10 | |  |
| damage | 5 | 12 | 9 | 4 | 3 | 11 | 8 | 4 | | 12 | | 1 | 14 | 7.5 | | 8 | |  |
| ACCT | 3 | 14 | 4 | 17 | 13 |  | 1 | 3 | | 4 | | 10 | 10 | 7.2 | | 7 | |  |
| possession | 8 | 11 | 14 | 10 | 4 | 8 | 3 | 6 | | 7 | | 5 | 3 | 7.2 | | 7 | |  |
| hooch | 11 | 1 | 7 | 6 | 7 | 5 | 9 | 7 | | 4 | | 5 | 5 | 6.1 | | 6 | |  |
| disobey | 6 | 15 | 3 | 2 | 5 | 10 | 1 | 8 | | 2 | | 2 | 8 | 5.6 | | 4 | |  |
| find | 14 | 8 |  | 3 | 10 | 8 | 3 | 2 | | 7 | | 5 | 4 | 5.8 | | 5 | |  |
| absent | 7 | 2 | 2 | 5 | 6 | 2 | 3 | 12 | | 3 | | 15 | 0 | 5.2 | | 3 | |  |
| smoking device | 2 | 6 | 1 | 8 | 1 | 1 | 3 | 10 | | 1 | | 5 | 6 | 4.0 | | 2 | |  |
| low mood | 1 | 7 | 4 | 1 | 2 | 3 | 7 | 1 | | 4 | | 3 | 2 | 3.2 | | 1 | |  |
| other | 0 | 0 | 0 | 0 | 0 | 0 | 0 | 0 | | 0 | | 0 | 1 | 0.1 | | 0 | |  |

Appendix D: Table two barriers and facilitators to the theory of change model

| Individual personal prisoner constructs | Prison Environment | Organisational elements | Barriers to good mental health in prison | Facilitating good mental health in prison |
| --- | --- | --- | --- | --- |
| Feeling safe  Empowerment  Personal Autonomy  Social anxiety  Trust  Empathy  Privacy  The ability to ask for help  Confidence  Coping strategy  Lonely  Detox  Clean/Decent  Outside pressures  Peer perspective and attitude  Debt  Setting goals  Drugs  Owing your own problems  Having a voice  Having someone to talk to  Peer support  Feeling isolated | Sudden changes to the regime  Changes to the amount of social time  Sleep and noise  Gangs  Intimidation  Segregation  Pending release  Bullying  Reducing In cell time  Lack of family contact  Lack of phones  Disruptions to workshop activity  Regime disruptions  Limited access to the gym and exercise  Violence | Financial limitations  Crisis management  Consistency of approach  Normalised responses to crisis  Organisation of the prison  Training for staff  Providing purposeful activity  Relationships between staff and prisoners  Staff perceptions and attitudes  Provision of resources  Food and diet  Level of staffing  Leadership  Provision of workshops and a variety of educational opportunities  Provision of interventions  Links to outside agencies to support drug and alcohol rehabilitation/ mental health/St Giles Housing | Sudden changes to the regime  Changes to the amount of social time  Sleep and noise  Gangs  Intimidation  Segregation  Bullying  Reducing In cell time  Lack of family contact  Lack of phones  Disruptions to workshop activity  Regime disruptions  Limited access to the gym and exercise  Violence | Feeling safe  Empowerment  Personal Autonomy  Trust  Empathy  Privacy  The ability to ask for help  Confidence  Coping strategy  Setting goals  Owing your own problems  Having a voice  Having someone to talk to  Peer support |

Appendix E: Table three change in wing by problem support mentor

| Original wing | Wing Movement | | | | | Total |
| --- | --- | --- | --- | --- | --- | --- |
|  | Wing D | Wing F | Wing G | Wing I | Wing J |  |
| Wing A | 0 | 0 | 0 | 1 | 0 | 1 |
| Wing D | 0 | 0 | 0 | 1 | 0 | 1 |
| Wing E | 1 | 0 | 0 | 0 | 1 | 2 |
| Wing G | 1 | 0 | 0 | 0 | 0 | 1 |
| Wing H | 0 | 0 | 1 | 0 | 0 | 1 |
| Wing J | 0 | 1 | 0 | 0 | 0 | 1 |
| C wing blue spur | 1 | 0 | 0 | 0 | 0 | 1 |
| Total | 3 | 1 | 1 | 2 | 1 | 8 |

Appendix F: Figure 5 Problems dealt with by problem support mentors


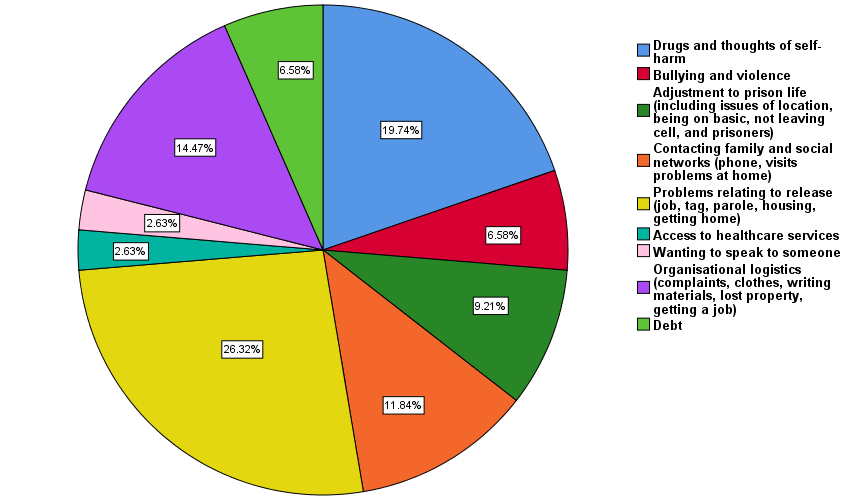


Appendix G: Table four case and controls baseline characteristics

| Demographic item |  | Cases  N=36 | Controls N=36 | P-value |
| --- | --- | --- | --- | --- |
| Age | Mean (SD),  Range  Valid cases | 35 (10.5)  23-67  N=36 | 35 (9.8),  24-67,  N=32 | 0.833 |
| Incentive and Earned privilege scheme N (%) | Basic  Standard  Enhanced  Valid cases | 0  7 (23.3)  23 (76.7)  N=30 | 4 (13.8)  12 (41.4)  13 (44.8)  N=29 | 0.014* |
| Attendance at previous accredited prison training course? N (%) | Yes  No  Valid cases | 20 (76.9)  6 (23.1)  N=26 | 9 (52.9)  8 (47.1)  N=17 | 0.101 |
| Offence category N (%) | Robbery  Conspiracy  Theft and Burglary  Fraud and money laundering  Possession/or supply of drugs  Murder  Endanger/violence/weapon  GBH  Driving offences  Fray | 4 (11.1)  1 (2.8)  2 (5.6)  6 (16.7)  10 (27.8)  2 (5.6)  0 (0)  9 (25)  1 (2.8)  1 (2.8) | 3 (8.3)  2 (5.6)  3 (8.3)  3 (8.3)  10 (27.8)  1(2.8)  8 (22.2)  5 (13.9)  1 (2.8)  0 (0) | 0.110* |
| Sentence length months, | Mean (SD)  Range  Valid Cases | 57.5 (29.6), 10-126  N=32 | 48.7 (23.2), 13-120  N=31 | 0.146 |

*Fisher’s exact test

Appendix H: Table five qualitative themes

| Thematic Category | Emerging Themes |
| --- | --- |
| Selection and Training | Personal qualities of a good problem-solving mentor |
|  | The Training Process |
|  | Ongoing supervision and intervention updates |
| The PST intervention | Reported benefits to the mentors themselves |
|  | Customer problems and benefits |
|  | Reducing impulsivity |
|  | The feeling of helping others |
|  | Peer support Vs Staff support |
|  | Perceived benefits to staff |
| Organisational Support and barriers to sustainability | Raising awareness and ‘buy-in’ from staff |
|  | Staff referrals to the intervention |
|  | The development of the role |

1. The higher the ranking the more severe the reported incident [↑](#footnote-ref-1)
2. Ranking of behavior by staff resulted in the following order from most to least severe (assault, fire, fight, threats, endangers, barricade, self-harm, under the influence, drugs, damage, ACCT and possession were equally ranked, hooch, disobey, find, absent, smoking devices, low mood and other). [↑](#footnote-ref-2)
